# Optimisation of enzymatic saccharification of wheat straw pre-treated with sodium hydroxide

**DOI:** 10.1038/s41598-021-02693-2

**Published:** 2021-12-01

**Authors:** Zhiquan Wang, Suqing Wu, Chunzhen Fan, Xiangyong Zheng, Wei Zhang, Deyi Wu, Xinze Wang, Hainan Kong

**Affiliations:** 1 School of Life and Environmental Science, Wenzhou University, Chashan, Wenzhou, 325035 People’s Republic of China; 2 School of Environmental Science and Engineering, Shanghai Jiao Tong University, Shanghai, 200240 Minhang People’s Republic of China

**Keywords:** Biocatalysis, Biogeochemistry, Enzymes

## Abstract

To enhance the reducing sugar yield in enzymatic hydrolysis, various factors (NaOH concentration, solid content and pre-treatment time) that affect the pre-treatment process were investigated and evaluated based on the reducing sugar yield of the subsequent enzymatic hydrolysis. The enzymatic hydrolysis was based on the cellulase from *Trichoderma reesi* ATCC 26921, the optimum NaOH pre-treatment conditions were an NaOH concentration of 1.0% (w/w), a solid content of 5.0% (w/v) and a pre-treatment time of 60 min. Various parameters that affect the enzymatic hydrolysis of wheat straw, including the solid content, enzyme loading, pH and hydrolysis time, were investigated and optimized through a Box–Behnken design and response surface methodology. The predicted optimum conditions for enzymatic hydrolysis were a solid content of 8.0% (w/v), an enzyme loading of 35 FPU/g substrate, a temperature of 50 °C, a pH of 5.3 and a hydrolysis time of 96 h. The experimental result showed that the maximum reducing sugar yield was 60.73% (53.35% higher than the wheat straw without NaOH pre-treatment), which is in accordance with the predicted conditions.

## Introduction

The development of clean, efficient, and renewable energy sources has reached a global consensus, including the greenhouse gas (GHG) emission reduction targets and energy supply security. Optimizing energy structure has also been an important part of China’s energy adjustment strategy^1^. The adjustment of energy structure and development of non-fossil energy and efficient use of fossil energy have been the main direction of China. Bioethanol, particularly which produced from lignocellulosic residues composed of cellulose (40–50%), hemicellulose (25–35%) and lignin (15–20%), is a promising fuel because of its GHG projected greenhouse gas (GHG) emission benefits^2,3^. Furthermore, lignocellulosic resources are considerably abundant, one promising way to convert abundant and renewable biomass materials to ethanol is an enzymatic hydrolysis process, which has been reported to exhibit high sugar yield, few by-products and low costs, but the structural properties of lignocellulose lead to the resistance of bioconversion^4^. However, this process is markedly hindered due to the complex and compact structures of the plant cell walls^5–7^. A pre-treatment step to break the lignin seal and disrupt the crystalline structure of cellulose is currently viewed as a critical step in the production of ethanol from lignocellulosic biomass, the internal structures of lignin were greatly altered after pretreatment, resulting in the separation of cellulose from lignin and the decomposition of lignin from large molecules with three-dimensional network structures to small molecules with linear structures^7,8^. Additionally, this step has a marked effect in terms of structural modifications to the lignocellulose material, which greatly benefits the enzymatic hydrolysis and subsequent steps^9–11^. Dilute NaOH pre-treatment, one of the most efficient pre-treatment methods, is more effective for agricultural residues and herbaceous crops. Enzymatic hydrolysis is a considerably sophisticated process that is greatly affected by a combination of controllable parameters, such as the solid content, hydrolysis time and enzyme loading, and these factors often interact with one another.

Response surface methodology (RSM) is a useful statistical technique for the modelling and optimisation of complex reaction processes^12–14^ and has already been successfully applied to the optimisation of medium and fermentation^15,16,17^. In this work, enzymatic hydrolysis efficiency and observations of the surface shape of wheat straw (WS) before and after NaOH pre-treatment using scanning electron microscopy (SEM) were used to investigate the effects and suitable values of the pre-treatment parameters. Furthermore, RSM using the Box-Behnken design (BBD) was exploited to identify the optimal conditions for reducing sugar (RS) production from NaOH-pre-treated WS through enzymatic hydrolysis. This was accomplished by analyzing the relationships among the parameters that affect the process.

## Materials and methods

### Raw material and enzyme

The raw material and enzyme were consistent with our previous studies^15^. The WS was harvested from the rural area around Tai Hu Lake and milled to pass through an 80-mesh screen (particle size of approximately 0.18 mm), and the commercial cellulase was from *Trichoderma reesi* ATCC 26921 (Celluclast 1.5 L). Same methods were operated to obtain the raw materials.

### NaOH pre-treatment

The process of NaOH pre-treatment mainly referred to our previous research^15^. Instead, the dried WS powder was pre-treated with different concentrations of NaOH solution (0.25–4.0%, w/w) with different solid contents (2.5–20%, w/v) and pre-treatment times (15–90 min).

### Enzymatic hydrolysis

#### Enzymatic hydrolysis of WS pre-treated with NaOH under different conditions

The enzymatic hydrolysis experiments were conducted in 50-mL DURAN glass bottles (SCHOTT, Germany) containing a total liquid volume of 20 mL and agitated on a rotatory shaker at 180 rpm for 96 h. The enzymatic hydrolysis conditions were a solid content of 4.0% (w/v), 0.4 g of dry pre-treated WS, 10 mL of liquid, a pH of 4.8 controlled with 50 mM citrate buffer, a duration of 96 h, a temperature of 40 °C and an enzyme loading of 30 FPU/g substrate. At the end of the process, the saccharification liquor was immediately decanted to the centrifugal tub, sealed with parafilm and centrifuged at 4000 rpm for 10 min. The supernatant was filtered through 0.45-µm membrane filters and maintained in 2-mL bottles at − 4 °C for later RS determination by high-performance liquid chromatography (HPLC).

#### Optimisation of enzymatic hydrolysis process

The enzymatic hydrolysis process of WS pre-treated with NaOH was performed exactly according to “Enzymatic hydrolysis of WS pre-treated with NaOH under different conditions” section. The solid content (2.0–8.0%, w/v), enzyme loading (10–35 FPU/g substrate), temperature (40–50 °C), pH (4.0–6.0) and time (12–96 h) were varied based on the Box–Behnken-designed experiments, and the experiments were carried out in duplicate.

#### Analytical methods

The cellulose, hemicellulose and lignin contents were determined based on the standard NREL procedure. A SEM JSM-740F (JEOL LTD, Japan) was used to observe the WS surface shape before and after the NaOH pre-treatment to indirectly investigate the effect of NaOH pre-treatment on the WS surface and the enzymatic hydrolysis process. The sugar samples were analysed by an HPLC instrument (LC-10AD, SHIMADZU, Kyoto, Japan) equipped with a refractive index detector (RID-10A, SHIMADZU, Kyoto, Japan). An Aminex HPX-87 P column (Bio-Rad, USA) with a safeguard column operated at 80 °C using pure-grade water as the mobile phase (0.6 mL/min) was utilized for the separation^15^. The RS yield was calculated according to the stoichiometric relationship represented by Eq. (1).

1 $${\text{Y}}_{{{\text{RS}}}} \left( \% \right) = {\text{C}}_{{{\text{RS}}}} /{\text{M}}_{{{\text{WS}}}} ,$$

where Y_RS_ is the actual RS yield, C_RS_ is the RS concentration in the saccharification liquid, and M_WS_ is the initial mass of the WS.

#### Experimental design and analysis

BBD method was used to analyze the effects of variables on RS yield (response) as described in Zhang et al.^15^. Five variables for BBD, consisting of six central points and three levels for each variable, i.e., − 1, 0, and + 1, was selected for the optimization, and a total of 46 runs were calculated. The range and levels of independent variables and coded values are presented in Table 1. Each experiment was performed in duplicate, and the mean values are reported.

**Table 1:** Independent variables and levels in the Box–Behnken design.

Independent variables	Symbols	Range and levels		
		− 1	0	1
Solid content (%, w/v)	*X*_1_	2.0	5.0	8.0
Enzyme loading (FPU/g substrate)	*X*_2_	10	22.5	35
Temperature (°C)	*X*_3_	40	45	50
pH	*X*_4_	4.0	5.0	6.0
Hydrolysis time (h)	*X*_5_	12	54	96

### Permission statement

We confirm that we have permission to collect wheat straw in Tai Lake by participating in the Major Science and Technology Program for Water Pollution Control and Treatment (2009ZX07101-015-003). Meanwhile, the wheat straw is not the specie at risk of extinction.

### Guideline statement

We confirm that the experimental research on wheat straw all comply with relevant institutional, national, and international guidelines and legislation in this study.

## Results and discussion

### Effect of NaOH pre-treatment operation variables on RS yield

**Figure 1:**
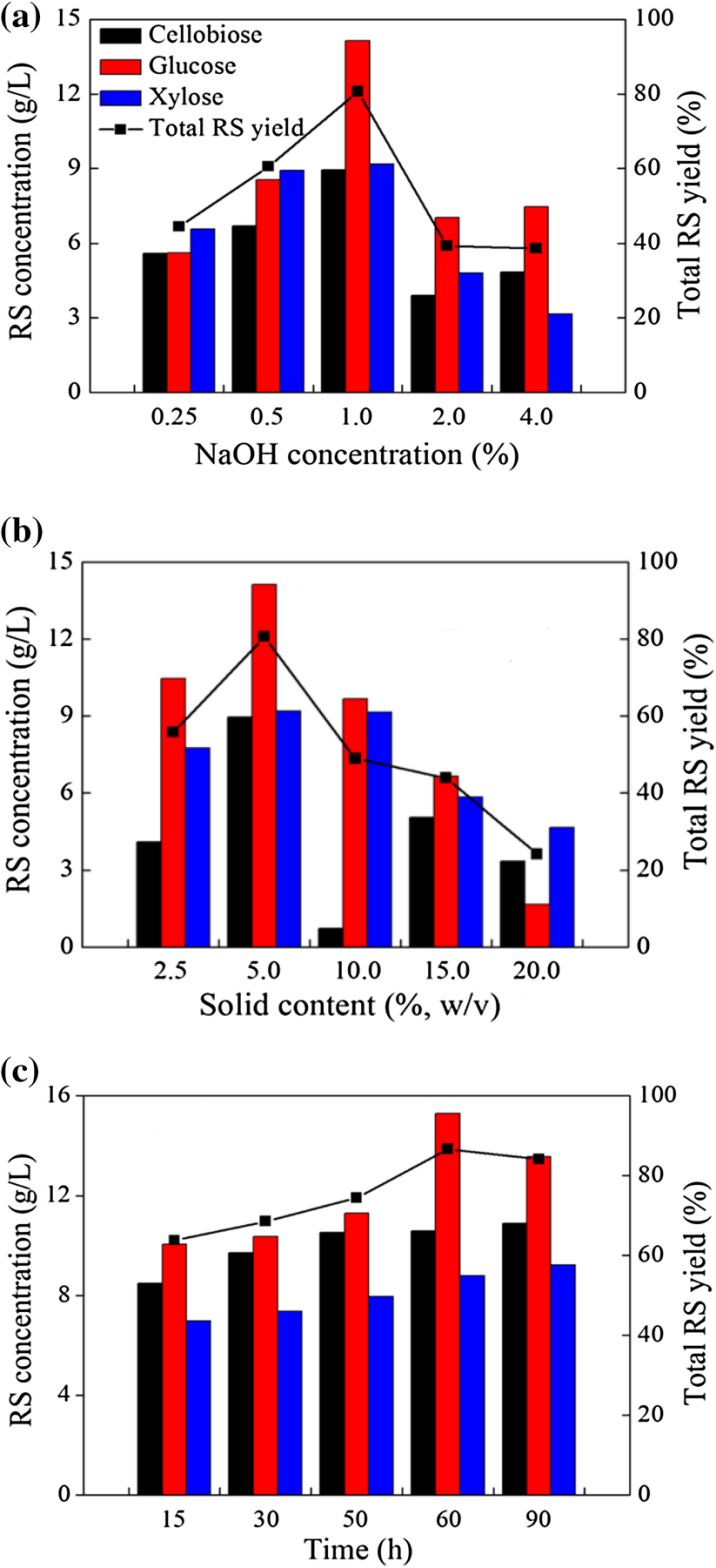
(**a**) RS yield after 96 h of the enzymatic hydrolysis of the WS pre-treated with different concentrations of NaOH ranging from 0.25% (w/w) to 4.0% (w/w). NaOH pre-treatment conditions: temperature, 121 °C; solid content, 5.0% (w/v); and time, 50 min; (**b**) RS yield after 96 h of the enzymatic hydrolysis of the WS pre-treated with 1.0% (w/w) NaOH at different solid contents ranging from 2.5% (w/v) to 20.0% (w/v). NaOH pre-treatment conditions: temperature, 121 °C; NaOH concentration, 1.0%; and time, 50 min; (**c**) RS yield after 96 h of the enzymatic hydrolysis of the WS pre-treated with 1.0% (w/w) NaOH for different times ranging from 15 to 90 min. NaOH pre-treatment conditions: temperature, 121 °C; NaOH concentration, 1.0%; and solid content, 5.0% (w/v).

The RS yield change in NaOH pre-treatment operation with a temperature of 121 °C, solid content of 5.0% (w/v) and pre-treatment time of 50 min is shown in Fig. 1a, the RS yield increased with an increase in the NaOH concentration and reached a maximum value of 80.65% when the NaOH concentration was 1.0% (w/w). A higher RS yield was not obtained with a further increase in the NaOH concentration to 4.0% (w/w). On the contrary, the RS yield markedly decreased. It has been reported that a long soaking in a high concentration of NaOH causes the dissolution of pentose, which leads to a reduction of RS recovery. In addition, high concentrations of NaOH can also increase the difficulty of washing away the alkalinity of the WS, which may also inhibit the latter enzymatic hydrolysis process. The RS yield change with a temperature of 121 °C, NaOH concentration of 1.0% (w/w) and pre-treatment time of 50 min is shown in Fig. 1b, the RS yield reached its maximal value at a solid content of 5.0% (w/v). A further increase in the solid content adversely affected the RS yield. The solid content played an important role in the pre-treatment process. A smaller solid content was associated with a relatively greater mass of NaOH that in contact with the same weight of WS. With a gradual increase in the solid content, the mixability of the mixture decreased, which resulted in a poor pre-treatment effect. Additionally, the RS yield decreased when the solid content was too low because much of the pentose was also dissolved. Thus, the maintenance of a suitable solid content in the pre-treatment process had a great significance on the increase in the RS yield. The RS yield change with a temperature of 121 °C, NaOH concentration of 1.0% (w/w) and solid content of 5.0% (w/v) is illustrated in Fig. 1c, the RS yield increased with an increase in the pre-treatment time and reached the maximal value of 86.63% with a pre-treatment time of 60 min. However, it started to slowly decrease as the pre-treatment time was further prolonged. Pre-treatment for a long time at a high temperature would also cause the dissolution of cellulose, which has a negative effect on the RS yield. Thus, to obtain a high RS yield, the optimal pre-treatment conditions were 1.0% NaOH, a solid content of 5.0% (w/v), and a pre-treatment time of 60 min.

**Figure 2:**
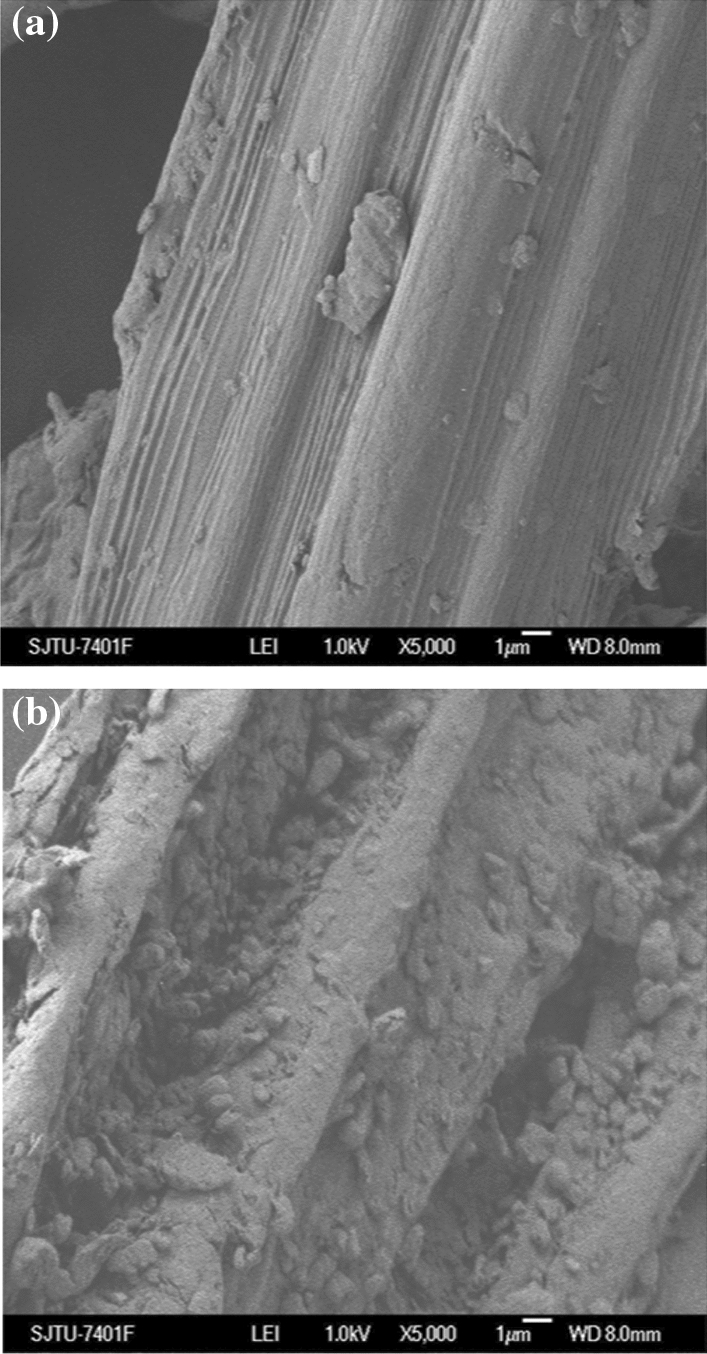
(**a**) SEM images of WS before pretreated with NaOH; (**b**) SEM images of WS after pretreated with NaOH under optimal conditions.

The composition change of WS after NaOH pre-treatment under the optimal pre-treatment conditions was shown in Table 2. According to the compositional analysis, the components of the WS after pre-treatment were as follows: 58.84% cellulose, 22.85% hemicellulose and 11.75% lignin. After the optimized NaOH pre-treatment, the cellulose composition was increased by 19.53%, whereas the lignin content was decreased by 13.98%. Unlike cellulose, the hemicellulose composition was changed from 21.51 to 22.85% which was not significant. NaOH pre-treatment provides effective delignification, and cellulose is more vulnerable to chemical pre-treatment than hemicellulose^18^. As the SEM photographs depict in Fig. 2a, the non-pre-treated WS had a smooth and ordered surface. However, the surface after pre-treatment with NaOH was rough and disordered, which is appeared to have cracks Fig. 2b. NaOH caused effective swelling, broke the bonds between lignin and carbohydrates, increased the internal surface of cellulose and decreased the degree of polymerisation and crystallinity, which disrupted the lignin structure and destroyed the compact structure. All these effects make the carbohydrates more accessible to enzymatic attack and may eventually improve the subsequent enzymatic hydrolysis efficiency^19^.

**Table 2:** The composition change of WS after NaOH pre-treatment.

Composition	% (w/w)	
	Unpretreated	NaOH pretreated
Cellulose	39.31	58.84
Hemicellulose	21.51	22.85
Lignin	25.73	11.75

### Interactive effects of the variables on RS yield

**Figure 3:**
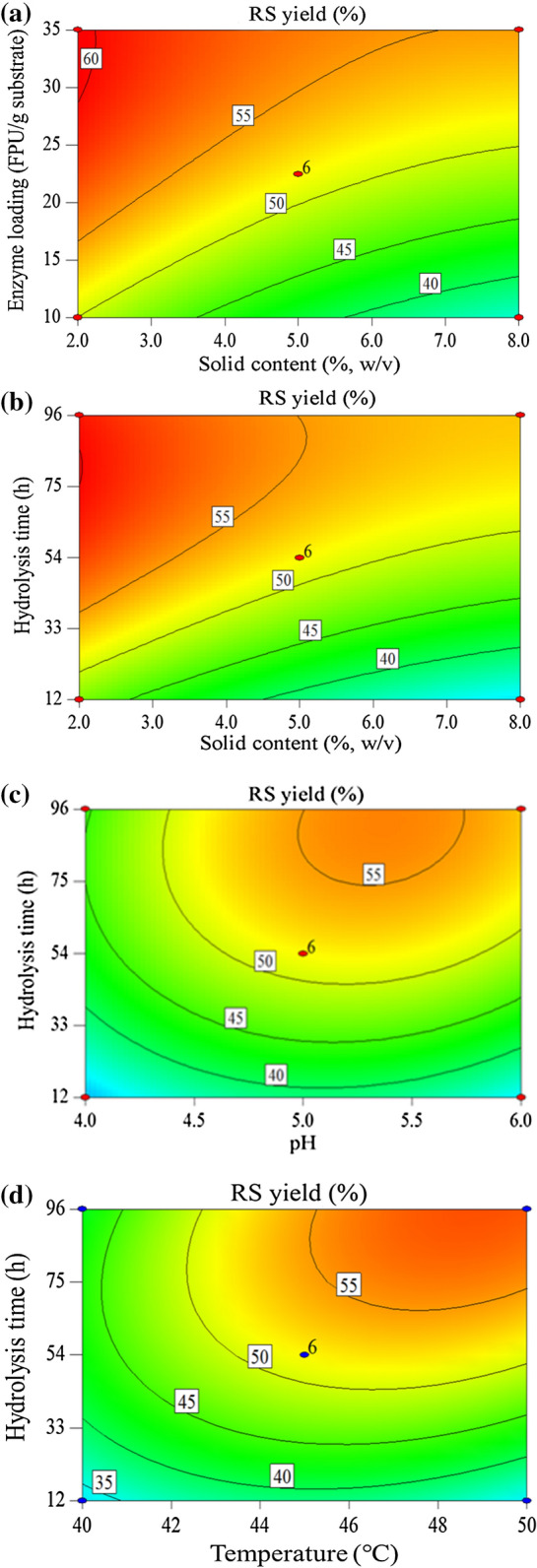
(**a**) Contour plots of the effects of the solid content and enzyme loading on the RS yield at an initial temperature of 45 °C, pH value of 5.0 and hydrolysis time of 54 h. (**b**) Contour plots of the effects of the solid content and hydrolysis time on the RS yield with an enzyme loading of 22.5 FPU/g substrate, a temperature of 45 °C and a pH value of 5.0. (**c**) Contour plots of the effects of the pH and hydrolysis time on the RS yield at an initial solid content of 5.0% (w/v), an enzyme loading of 22.5 FPU/g substrate and a temperature of 45 °C. (**d**) Contour plots of the effects of the temperature and hydrolysis time on the RS yield at an initial solid content of 5.0% (w/v), an enzyme loading of 22.5 FPU/g substrate and a pH value of 5.0.

In this study, contour plots were used to investigate the interactive effects of two independent variables on the response while the other two variables were maintained constant at the middle level. As shown in Fig. 3a, the mixability of the hydrolysate greatly decreased with an increase in the solid content when the enzyme was maintained at the low level (10 FPU/g substrate), and a limited amount of enzyme could not sufficiently adsorb to the straw, resulting in a marked decrease in the RS. The RS released by the enzymatic hydrolysis had a feedback inhibition effect on the activity of the enzyme, which further hindered the reaction^20^. However, when the enzyme loading was maintained at a high level (35 FPU/g substrate), an increase in the solid content had a slight effect on the RS yield because there was sufficient enzyme to quickly hydrolyse the cellulose into RS. A change in the enzyme loading had a greater effect when the solid content was maintained at a high level (8.0%, w/v) than when it was held at a low level (2.0%, w/v). In addition, a higher solid content may be desirable because it can increase the ethanol concentration in the subsequent fermentation process, which is crucially important for cost reduction^21,22^. Figure 3b shows the interactive effect of the solid content and hydrolysis time on the RS yield. It can be deduced that the RS yield increased markedly with an increase in the hydrolysis time, particularly when the solid content was maintained at a high level (8.0%, w/v). It can also be inferred that the RS yield reached a considerable value within the first 12 h, particularly when the solid content was held at a low level (2.0%, w/v). Figure 3c shows the interactive effect of the pH and hydrolysis time on the RS yield. The RS yield increased with an increase in the pH value over a certain range. A further increase in the pH had an adverse effect on the ethanol yield. The trend in the RS yield verified that the pH has a positive linear effect but a negative quadratic effect on the RS yield at the 5% level. The change in pH can affect the dissociation degree of a critical group at the active site, which would affect the interaction between the enzyme and the substrate. Figure 3d shows the effect of temperature and hydrolysis time on the RS yield, the interaction between temperature and hydrolysis time was significant (P < 0.05) and a positive effect was obtained with the higher temperature and longer hydrolysis time.

### Validation of the model

The model predicted a maximum RS yield of 64.44% at the optimal conditions of a solid content of 8.0% (w/v), an enzyme loading of 35 FPU/g substrate, a temperature of 50 °C, a pH value of 5.3 and a total hydrolysis time of 96 h. The experiments used to validate the optimised parameters and the predicted RS yield were performed in duplicate. The mean experimental RS yields of non-pre-treated and optimised NaOH-pre-treated WS were found to be 60.73% and 7.38%, respectively. Additionally, the RS yield obtained under the optimised conditions was improved by 8.86% compared with the un-optimised process.

In this study, the adequate precision value was 38.730, which is an adequate signal. The low value obtained for the coefficient of variation (CV, CV = 2.87%) indicated that the experiments were precise and reliable. The experiments used to validate the optimised parameters and the predicted RS yield were performed in duplicate. The RS yield of the optimised NaOH-pre-treated WS was in accordance with the predicted RS yield, which confirmed the accuracy of the developed model. The NaOH-pre-treated WS greatly enhanced the final RS yield. The RS reached the maximum concentration of 48.58 g/L, and the yield (60.73%, approximately 1.21 g/g substrate) achieved in this study was comparable with the results reported of 0.35 g/g TS dry biomass^23^. Furthermore, the accurate prediction of model can avoid excess initial addition of enzyme and cellulose, which significantly reduces the costs in the subsequent fermentation process.

## Conclusion

The NaOH pre-treatment of WS was successful for the removal of lignin and enhancement of the final RS yield. The optimal conditions for the NaOH pre-treatment were a NaOH concentration of 1.0% (w/w), a solid content of 5.0% (w/v), and a pre-treatment time of 60 min. Additionally, the optimal conditions for the enzymatic hydrolysis of the optimised NaOH-pre-treated WS were a solid content of 8.0% (w/v), an enzyme loading of 35 FPU/g substrate, a temperature of 50 °C, a pH of 5.3 and a hydrolysis time of 96 h. In the validation experiments, the mean experimental RS yield was found to be 60.73%, which was in accordance with the predicted yield (64.44%). Additionally, the RS yield was increased by 53.35% compared to that obtained with the non-NaOH-pre-treated WS under the same optimal hydrolysis conditions. The Box-Behnken design and the developed model can be used to navigate the designed space and predict the bioprocesses of the enzymatic hydrolysis system.
